# Robotic partial splenectomy: a new standardized approach

**DOI:** 10.1007/s00595-025-03140-9

**Published:** 2025-09-22

**Authors:** Mohamed Amine Tormane, Ambroise Ravenet, Francesco Carenini, Alexandre Lecis, Tarek Kellil, Tullio Piardi

**Affiliations:** 1 Department of HBP Surgery, Simone Veil Hospital, 101 Av. Anatole France, 10000 Troyes, France; 2 Reims Champagne-Ardennes University, 51 Rue Cognacq-Jay, 51100 Reims, France; 3 Department of Vascular Surgery, Simone Veil Hospital, 101 Av. Anatole France, 10000 Troyes, France

**Keywords:** Partial splenectomy, ICG, Embolization

## Abstract

The treatment of benign splenic diseases is trending toward a conservative approach, especially for young patients, due to the importance of preserving as much splenic parenchyma as possible. Minimally invasive partial splenectomy (PS), which has become the gold standard for managing benign splenic conditions, is generally associated with low morbidity and near-zero mortality. Herein, we describe a standardized and safe technique to perform this procedure. This new approach combines preoperative selective distal embolization of the splenic artery with indocyanine green (ICG)-guided robotic partial splenectomy using the technique of negative staining as applied in liver surgery.

**Supplementary Information:**

The online version contains supplementary material available at 10.1007/s00595-025-03140-9.

## Introduction

Minimally invasive splenectomy has become the gold standard for splenic diseases such as hematological disorders, cystic masses, and benign or malignant tumors of the spleen [1–3]. Adopting a conservative approach can help prevent the complications associated with total splenectomy, especially in young people who would be at risk of serious infections because of impaired immune defenses [4]. This approach includes non-surgical treatments such as splenic artery embolization, thermoablation, and surgical management with partial or subtotal splenectomy [5, 6]. Partial splenectomy (PS) is the preferred procedure for treating splenic lesions and reducing splenic size, while maintaining organ function [7]. Although this technique is performed widely, only a few cases of robotic PS have been reported and there is no standardized technique for PS described in the literature [8, 9]. Although case series show low morbidity and almost zero mortality rate for this procedure, it must be as safe as possible, especially as it is generally performed on young patients [10, 11]. The introduction of the Firefly real-time near-infrared imaging system into the Da Vinci platform has further incentivized the use of indocyanine green (ICG) [12, 13]. In pancreatic surgery, ICG is used to confirm the vascularization of the spleen during Warshaw’s procedure [14]. We describe a new safe approach combining selective splenic arterial embolization and ICG-guided robotic PS using the technique of negative staining, similar to that used in liver surgery, to visualize the line of spleen transection.

### Patients’ characteristics

**Fig. 1:**
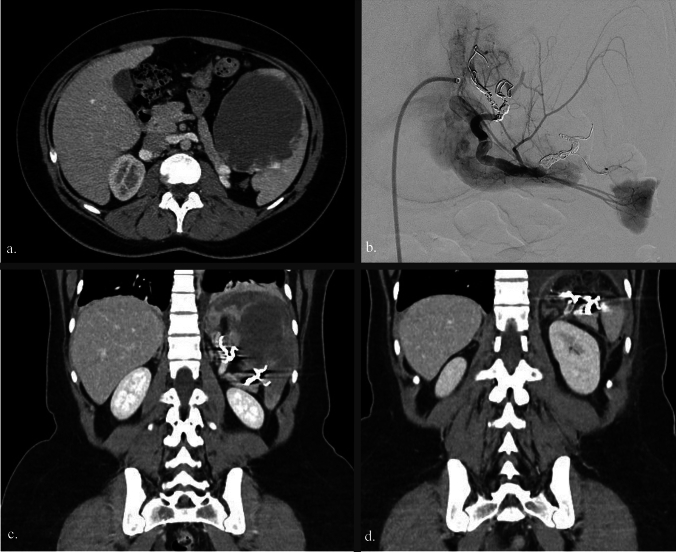
Preprocedural computed tomography (CT) scan (**a**); embolization of two branches of the splenic artery (**b**); post-embolization CT scan (**c**); postoperative CT scan (**d**)

Two young patients, a 19-year-old man and an 18-year-old woman, presented with enlarging splenic cysts, discovered incidentally or with noted progressive growth. Both patients reported pain in the left hypochondrium, but had unremarkable laboratory results, except for a low platelet count. Imaging examinations revealed large cystic lesions in the upper pole of the spleen in both patients. A computed tomography (CT) scan for the first patient showed a well-defined, fluid-dense cystic lesion (10 cm) without enhancement (Fig. 1a), while a magnetic resonance imaging (MRI) for the second patient identified a multiloculated cystic mass (11 cm) with high signal intensity on T2-weighted images and slight contrast uptake along the anterior wall, suggesting possible hemorrhagic foci. These findings were consistent with a cystic splenic lymphangioma or splenic hemangioma.

### Therapeutic procedure

**Fig. 2:**
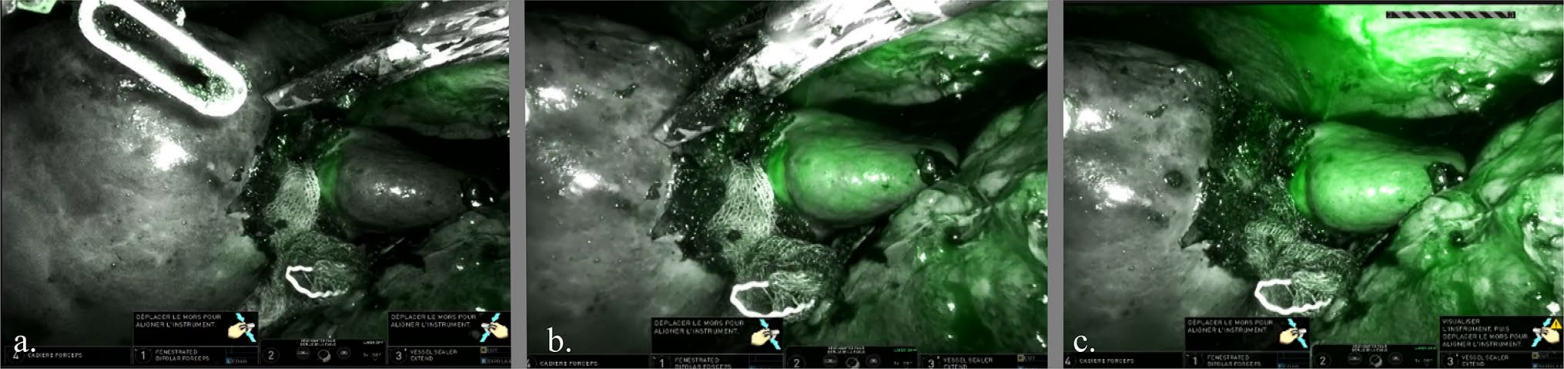
Intraoperative view with Firefly mode at 15 s (**a**), 35 s (**b**), and 50 s (c)

(I) Preoperative selective splenic artery embolization was performed in a hybrid operating room under general anesthesia. The patient was placed in the supine position and a femoral artery puncture was performed. Catheterization of the celiac trunk and then the splenic artery was carried out, advancing into the segmental branches of the spleen using a PROGREAT™ microcatheter for precise navigation. Arteriography assessed splenic segmentation, enabling selective embolization of the superior and middle territories while preserving the integrity of the inferior territory. Embolization was achieved with TERUMO coils AZUR™ CX, ensuring optimal occlusion. The final outcome was satisfactory, with preservation of the inferior pole of the spleen (Fig. 1b and video). A post-embolization abdominal scan confirmed good inferior splenic vascularization (Fig. 1c). Surgery was performed 7 days after the embolization. Between embolization and surgery, the patients reported experiencing no pain and there were no signs of an inflammatory response.

(IIa) Robot-assisted partial splenectomy (RAPS): under general anesthesia, the patient was placed in the French position, and the RAPS procedure was carried out via five ports placed in the upper abdomen (video). To reach the rear omental cavity, the left colonic flexure was pulled down, and the pancreas and spleen were exposed by incising the gastrocolic ligament using an ultrasonic scalpel.

(IIb) ICG was then used to highlight the vascularized part of the spleen and delineate the parenchymal demarcation line as “negative staining”. The intraoperative protocol included giving an intravenous (IV) injection of 0.5 mg/kg ICG and alternating between the white light and near-infrared channels on the console of the Da Vinci platform to define the line of parenchymal transection (Fig. 2). The splenic hilum was exposed after dividing the gastrosplenic ligament, allowing control of the splenic artery, which was placed on a vascular loop to allow handling. (For hilar tumors, and even more so when the splenic vessels divide late, we perform parenchymotomy first, which can be achieved safely after preoperative embolization, whereas for more peripheral tumors, we begin by skeletonizing and dividing the splenic vessels before proceeding with the parenchymotomy). Splenic parenchymal transection was then performed using a mechanical crushing device with two bipolar forceps: a curve grasper and a fenestrated grasper. This method combined clamp crushing with continuous aspiration and irrigation to prevent carbonization of the bipolar forceps jaws. We also used a thermofusion device for parenchymal transection and to divide the splenodiaphragmatic ligament. After the spleen was completely exposed, the splenic vein was divided using a vascular stapler for optimal hemostasis. The procedure was facilitated by puncture and aspiration of the cystic mass, and the splenic margin was treated with hemostatic mesh. A final vascularization check was performed using ICG.

At the end of the procedure, a drain was placed near the transection margin to monitor postoperative hemorrhage. The removed part of the spleen was placed in a specimen bag and extracted through an extended umbilical incision (3 cm). Surgery was completed successfully in both patients. The operative time for each patient was 120 min and 150 min, respectively, and the blood loss was 100 ml and 200 ml, respectively. The first patient’s postoperative platelet count was 200 G/L but the second patient’s was 1020 G/L, with a follow-up count of 500G/L on postoperative day (POD) 45, allowing for the discontinuation of anti-thrombotic medication. Abdominal CT scan done on POD 5 showed good vascularization of the remaining spleen (Fig. 1d). The patients were discharged the same day. Vaccinations were administered as a precautionary measure before and after surgery in accordance with French recommendations for patients with asplenia. Histopathology confirmed no evidence of malignancy. During 18 months of follow-up, the patients remained in good health with no recurrence of pain and no thrombocytosis that would have required the introduction of aspirin.

## Discussion

The combination of preoperative selective distal embolization, followed by RAPS with intraoperative ICG imaging, is a novel strategy to treat patients with large splenic cysts. Mini-invasive splenectomy has become the gold standard because of its advantages over open surgery, including less pain, faster recovery, less blood loss, and minimal scarring [2, 10]. Originally, total splenectomy was the only procedure performed, which was associated with the same postoperative risks as open surgery, namely: overwhelming post-splenectomy infection (OPSI), thrombocytosis, thromboembolic events, and pulmonary hypertension [15]. In recent years, preserving the immune function of the spleen became the cornerstone, especially in young patients who usually had benign lesions of the spleen diagnosed. Studies have found that preserving more than 25% of the spleen tissue can protect this function [16]. This led to the inception of PS, which is based on the splenic segmental blood supply. The splenic artery branches at the splenic hilum into different branching patterns [17]. The main challenges of PS include anatomical identification of the splenic hilum vasculature divisions, accurate identification of the blood supply to the previously preserved spleen parenchymal territory, and careful hemostasis during splenic transection.

There is no standardized technique to control potential bleeding during the transection of the parenchymal spleen, although several procedures, such as radiofrequency or temporary occlusion of proximal splenic artery with clamps, have been described [18, 19]. Moreover, with the advent of thermofusion technology, it is possible to start parenchymotomy immediately, without performing prior vessel dissection [20]. Preoperative splenic artery embolization has been performed before total laparoscopic splenectomy for massive splenomegaly [21, 22]. Segmental ischemia can be induced by embolizing the arterial branches corresponding to the affected splenic segment, being the upper and middle branches in our two patients. This allows the desired part of the spleen to be removed safely with a lower risk of intraoperative bleeding. This step also reduces intraoperative blood loss, enhances surgical precision, and minimizes overall operative morbidity. We performed surgery 7 days after embolization on an empirical basis to obtain satisfactory delineation. In fact, the timing between partial embolization and PS varies from 0 to 28 days depending on the team [23–25]. Nevertheless, based on studies about splenic embolization in trauma, the complication rate of the embolization itself (for abscess or cyst) is acceptable [26] and allows us to avoid combining the two procedures and obtain delimitation. Vasilescu et al. reported the first case of RAPS in 2010 [7], since when robotic surgery has become a superior minimally invasive technique and shows great promise in the treatment of various diseases, including splenic surgery. In comparison with laparoscopic surgery, robotic surgery significantly reduces intraoperative blood loss and vascular dissection time and allows for better evaluation of the splenic remnant volume [9]. There is no significant difference in postoperative hospital stay between robotic and laparoscopic surgery. Depending on the case, it may be necessary to ligate the main trunk of the splenic vein. Similar to the Warshaw procedure, this ligation may cause left-sided portal hypertension, although it has been shown that this left-sided portal hypertension associated with the Warshaw procedure has no long-term clinical consequences [27]. The use of ICG in RAPS offers a real-time assessment of splenic vascularization and ischemic zones (Fig. 2). This imaging technique allows the surgical team to delineate the ischemic regions of the spleen that need to be removed, confirm the vascular integrity of the remaining splenic tissue to prevent postoperative necrosis, guide precise margins for resection, and ensure optimal preservation of functional splenic parenchyma. Vaccination is still a subject of debate, particularly with splenic embolization, where there is a tendency to no longer vaccinate patients [28]. There are no recommendations regarding partial splenectomy, but as a precautionary measure, we administer vaccinations before and after surgery in accordance with the French recommendations for patients with asplenia.

## Conclusion

RAPS offers advantages in the treatment of benign splenic diseases by preserving the immunological function of the spleen. Selective preoperative embolization and the use of ICG can reduce the complexity of the procedure and make it safer. This multidisciplinary procedure underscores the evolution of splenic surgery towards minimally invasive, organ-sparing techniques, especially for young patients who will benefit from the preservation of long-term splenic function. Although this technique appears feasible, it should be studied in larger patient series and in a comparative manner before it can be officially recommended.

## Supplementary Information

Below is the link to the electronic supplementary material.

Supplementary file1 (MP4 65381 KB)
